# The Effects of Different Fine Recycled Concrete Aggregates on the Properties of Mortar

**DOI:** 10.3390/ma8052658

**Published:** 2015-05-18

**Authors:** Cheng-Chih Fan, Ran Huang, Howard Hwang, Sao-Jeng Chao

**Affiliations:** 1 Institute of Materials Engineering, National Taiwan Ocean University, No. 2 Pei-Ning Road, Keelung 20224, Taiwan; E-Mail: chengchihfan@gmail.com; 2 Department of Harbor and River Engineering, National Taiwan Ocean University, No. 2 Pei-Ning Road, Keelung 20224, Taiwan; E-Mail: ranhuang@ntou.edu.tw; 3 Graduate Institute of Architecture and Sustainable Planning, National Ilan University, No.1, Sec. 1, Shen-Lung Road, I-Lan 26047, Taiwan; E-Mail: hmhwang@niu.edu.tw; 4 Department of Civil Engineering, National Ilan University, No.1, Sec. 1, Shen-Lung Road, I-Lan 26047, Taiwan

**Keywords:** fine recycled concrete aggregate, recycled concrete aggregate, recycled aggregate, compressive strength, ultrasonic pulse velocity, mortar

## Abstract

The practical use of recycled concrete aggregate produced by crushing concrete waste reduces the consumption of natural aggregate and the amount of concrete waste that ends up in landfills. This study investigated two methods used in the production of fine recycled concrete aggregate: (1) a method that produces fine as well as coarse aggregate, and (2) a method that produces only fine aggregate. Mortar specimens were tested using a variety of mix proportions to determine how the characteristics of fine recycled concrete aggregate affect the physical and mechanical properties of the resulting mortars. Our results demonstrate the superiority of mortar produced using aggregate produced using the second of the two methods. Nonetheless, far more energy is required to render concrete into fine aggregate than is required to produce coarse as well as fine aggregate simultaneously. Thus, the performance benefits of using only fine recycled concrete aggregate must be balanced against the increased impact on the environment.

## 1. Introduction

The practical use of recycled concrete aggregate produced by crushing concrete waste reduces the consumption of natural aggregate as well as the amount of concrete waste that ends up in landfills. The crushing of concrete waste produces coarse recycled concrete aggregate (CRCA) and fine recycled concrete aggregate (FRCA), as defined by particle size. A number of studies [1,2,3,4,5,6,7,8,9,10,11] have shown that CRCA can be used as a replacement for coarse natural aggregate in structural concrete; however, relatively little research has been conducted on the application of FRCA in structural concrete [11,12,13,14,15].

Several studies [11,12,13,14,15,16,17,18,19] have used laboratory crushers for the crushing concrete of waste to produce FRCA. Test results of these materials reveal that the FRCA produced using this crushing process leaves a large amount of cement paste attached to the surface of FRCA, which can have a detrimental effect on the material properties. Thus, researchers have shifted their attention to the influence of production process on the properties of the resulting FRCA.

Lee [20] investigated two discrete crushing processes using a jaw crusher and an impact crusher to obtain two types of FRCA: RF-A and RF-B, with specific gravity values of 2.39 and 2.28 and water absorption of 6.59% and 10.35%, respectively. Various quantities of fine natural aggregate (FNA) were then replaced with the two types of FRCA, whereupon the resulting mortars were tested. The mortar in which FNA was replaced entirely by RF-A presented higher density and greater compressive strength than did the samples made entirely with RF-B. These results also indicate that the water absorption of FRCA influences the properties of the mortar, particularly at higher replacement ratios.

Sim and Park [21] applied advanced recycling methods to the production of FRCA with a specific gravity of 2.28 and water absorption of 6.45%. They replaced various proportions of FNA with FRCA and tested the resulting concrete specimens. Compressive strength was shown to decline with an increase in the replacement ratio of FRCA. When the replacement ratio reached 100%, the compressive strength of the mortar at 28 days was approximately 33% lower than that of the original samples and all specimens with over 60% FNA replacement presented a significant drop in compressive strength.

Florea and Brouwers [22] investigated the influence of concrete crushing method on the particle size distribution and density of recycled concrete aggregate (RCA). As a standard, they adopted concrete with compressive strength of 60 MPa at 91 days, to which they applied three methods for the crushing of concrete: (1) RC-1 refers to RCA from concrete waste that was passed through a jaw crusher just once before being screened; (2) RC-2 refers to RCA that passed through a jaw crusher ten times before being screened; and (3) RC-3 refers to RCA produced from three consecutive crushing processes using the Smart Crusher SC 1, designed specifically for concrete waste. RC-3 presented the optimal particle size distribution, between 125 μm and 200 μm, with a density of 2.50 g/cm^3^, which increased with the size of the particles. RC-3 particles between 2 mm and 4 mm in size had a density of 2.61 g/cm^3^. They concluded that optimizing the crushing method could enhance the quality of the resulting RCA.

Ulsen *et al.* [23] produced a variety of FRCAs by crushing recycled aggregates smaller than 19 mm using a jaw crusher in conjunction with a vertical shaft impact (VSI) crusher at various rotational speeds: (1) CDW-sand refers to FRCA produced using a jaw crusher prior to screening; (2) VSI-55 refers to FRCA produced using a jaw crusher followed by a VSI crusher at 55 m/s prior to screening; (3) VSI-65 refers to FRCA produced using a jaw crusher followed by a VSI crusher at 65 m/s prior to screening; and (4) VSI-75 refers to FRCA produced using a jaw crusher followed by a VSI crusher at 75 m/s prior to screening. Their results indicate that the rotational speed of the VSI crusher had no effect on the particle shape or particle size distribution of the FRCA; however, it did affect water absorption and porosity. The water absorption of CDW-sand, VSI-55, VSI-65, and VSI-75 were 12%, 9%, 8.1%, and 7%, respectively, whereas the porosity percentages were 11.9%, 6.9%, 5%, and 6%, respectively.

Song and Ryou [24] introduced a washing stage to the production of FRCA using a combination of chemical and physical processes. The washing process had the following effects: water absorption dropped from 5.8% to 1.92%; the ratio of absolute volume increased from 62.3% to 65.1%; and impurity content dropped from 0.46 to 0.18%. Clearly, this washing process can enhance the physical properties of the resulting FRCA.

Koshiro and Ichise [25] employed a heat grinder system for the processing of concrete waste from a demolished building, which resulted in FRCA with density of 2.57 g/cm^3^ and water absorption of 2.52%. Their results demonstrate the efficacy of heat grinder systems in the production of high-quality FRCA suitable for the structure of new buildings.

Clearly, the methods used in the processing of concrete waste influence the quality of the resulting FRCA. Most previous studies have obtained FRCA produced under laboratory conditions or using the methods typically employed in large-scale recycling facilities, in which CRCA and FRCA are produced simultaneously. In this study, we obtained FRCA from a recycling facility in Yilan, Taiwan, which using a crushing process that produces only fine recycled concrete aggregate. We then prepared and tested specimens using a variety of mix proportions to determine the influence of production process and FRCA proportion on the properties of the resulting mortar, which is a constituent of concrete.

## 2. Experimental

### 2.1. Materials

**Figure 1:**
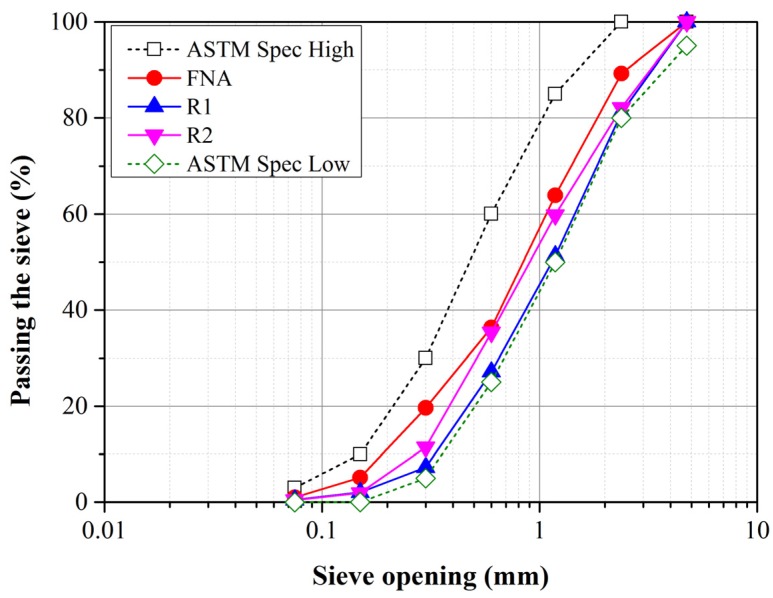
Particle size distribution curves of FNA, R1 and R2 (ASTM C33-13 [26]).

This study employed Type I Portland cement and FNA comprising clay slate and river sand, which was processed in a gravel plant. Figure 1 presents the particle size distribution of the FNA and Table 1 lists the basic physical properties.

**Table 1:** Physical properties of FNA, R1 and R2.

Physical properties	FNA	R1	R2
Saturated surface dry density (kg/m^3^) (ASTM C128-12 [27])	2653	2347	2404
Water absorption (%) (ASTM C128-12 [27])	1.3	8.9	6.6
Fineness modulus (ASTM C136-14 [28])	2.9	3.3	3.1

**Figure 2:**
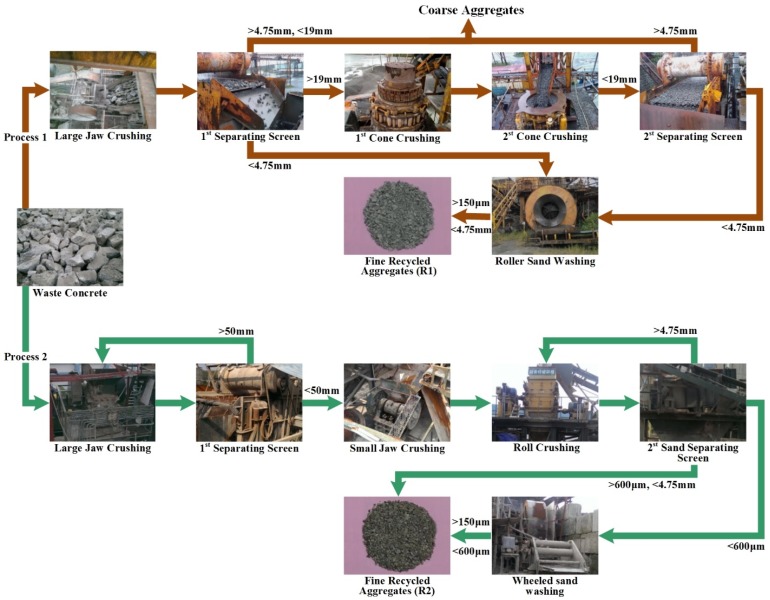
Processes used in the production of fine recycled concrete aggregates.

Figure 2 illustrates two processes commonly used for the production of fine recycled concrete aggregate. The first process produces coarse and fine aggregates simultaneously by crushing waste concrete with a large jaw crusher and then separating the resulting aggregate using a vibrating screen. Aggregate larger than 19 mm in diameter is sent through two cone crushers, after which the product is separated using a vibrating screen. Aggregate between 4.75 mm and 19 mm is transported to a coarse aggregate stockpile area. Aggregate smaller than 4.75 mm is sent to a roller sand washer to wash away sludge smaller than 150 μm. The resulting product is fine aggregate (ranging in size from 150 μm to 4.75 mm), which is stored in a fine aggregate stockpile area. The FRCA produced by this process is denoted as R1, the properties or which are presented in Figure 1 and Table 1.

The other method employed in this study produces only fine aggregate using multiple processes. Concrete waste first undergoes crushing in a large jaw crusher, whereupon the resulting aggregate is separated using a vibrating screen. Fragments exceeding 50 mm are sent back to the large jaw crusher to undergo repeated crushing, and this cycle is repeated until all of the aggregate is less than 50 mm. The resulting material is then crushed using a small jaw crusher and a roll crusher and separated using a vibrating screen. Aggregate larger than 4.75 mm is returned to the roll crusher until it is all smaller than 4.75 mm. Aggregate between 600 μm and 4.75 mm is sent to a fine aggregate stockpile; whereas aggregate smaller than 600 μm is sent to a wheeled sand washer for the removal of sludge smaller than 150 μm. The remaining aggregate between 150 μm and 600 μm is sent to the fine aggregate stockpile. The second type of FRCA produced using the above-mentioned process is denoted as R2, the basic properties of which are also presented in Figure 1 and Table 1.

### 2.2. Mix Proportions

Table 2 presents the mix proportions adopted for the mortar in this study. The water/cement ratios were set at 0.35 and 0.55; however, the mortar from the former mix displayed poor flowability; thus a superplasticizer (0.5% of the weight of the cement) was added to increase flowability. The replacement level of FNA by R1 and R2 were set at volume fraction of 0%, 25%, 50%, and 100%. As shown in Table 2, the weight ratio for cement and FNA in the control groups is 1:2. Because the densities of R1 and R2 are different from that of FNA, the weights of R1 and R2 in other groups are different from that of FNA used in the control groups, as shown in Table 2.

**Table 2:** Mix proportions of mortar specimens.

Mix notation	W/C ratio	FRCA content (%)	Mix proportions (kg/m^3^)					
			Water (kg)	Cement (kg)	FNA (kg)	R1 (kg)	R2 (kg)	Superplasticizer (kg)
AControl	0.35	0	245	700	1400	4
A25R1	0.35	25	245	700	1050	310	4
A50R1	0.35	50	245	700	700	619	4
A100R1	0.35	100	245	700	1239	4
A25R2	0.35	25	245	700	1050	317	4
A50R2	0.35	50	245	700	700	634	4
A100R2	0.35	100	245	700	1269	4
BControl	0.55	0	340	620	1240	-
B25R1	0.55	25	340	620	930	274		-
B50R1	0.55	50	340	620	620	548		-
B100R1	0.55	100	340	620		1097		-
B25R2	0.55	25	340	620	930		281	-
B50R2	0.55	50	340	620	620		562	-
B100R2	0.55	100	340	620			1124	-

### 2.3. Fabrication of Specimens

The FRCA used in this study presents a higher water absorption than does FNA. We therefore applied pre-wetting to R1 and R2 sample for 24 h. We then adjusted the measurement of surface moisture (ASTM C70-13 [29]) prior to mixing in order to achieve saturated-surface-dry (SSD) conditions using the water compensation method. Mixing was performed according to set proportions and the resulting mortar for each mixture was used to produce the following: six cylindrical specimens with a diameter of 100 mm and height of 50 mm, three cylindrical specimens with a diameter of 100 mm and height of 200 mm, four 285 × 25 × 25 mm prismatic specimens, and nine 50 × 50 × 50 mm cubic specimens. After casting, the specimens were covered with plastic sheeting to prevent evaporation. The samples stood in the laboratory for 24 h before being de-molded and held in saturated lime water for curing at a mean temperature of 23 ± 2 °C until the time of testing.

### 2.4. Testing

Various tests were performed to characterize the attributes of the mortar, including flow tests, absorption tests, density tests, drying shrinkage tests, compressive strength tests, and ultrasonic pulse velocity (UPV) tests.

Flow testing was performed in accordance with ASTM C1437-13 [30]. Freshly mixed mortar was poured into a flow mold with a bottom diameter of 100 mm (D_0_) on a flow table. The flow mold was then lifted off and the flow table was vibrated. The mean diameter (D_a_) of the resulting flow was calculated using results obtained after conducting the test four times, as follows:

(1) Flow (%) = (D_a_ − D_0_)/D_0_ × 100 (%)

Density testing was performed using cylindrical specimens with a diameter of 100 mm and height of 50 mm in accordance with ASTM C642-13 [31]. The specimens were weighed at 28 days in SSD condition (W_s_). The specimens were weighed while suspended in boiling water 5 h (W_a_) and again after being removed from the water that had cooled to 25 °C (W_b_). The density of the specimens was calculated using the following formula.

(2) Density (kg/m^3^) = [W_s_/(W_b_ − W_a_)] × 1,000 (kg/m^3^)

Absorption testing was performed using cylindrical specimens with a diameter of 100 mm and height of 50 mm, in accordance with ASTM C642-13 [31]. Specimens at 28 days were first placed in an oven at 105 ± 5 °C and dried until achieving constant weight (W_d_). They were then soaked in water to achieve the SSD condition before being weighted (W_s_). The water absorption was calculated using the following formula:

(3) Absorption (%) = (W_s_ − W_d_)/W_d_ × 100 (%)

Drying shrinkage was tested on 285 × 25 × 25 mm prismatic specimens in accordance with ASTM C596-09 [32]. We first measured the initial length (L_i_), which is the difference between the comparator reading of the specimen and the reference bar at 3 days. The specimens were then placed in a chamber under relative humidity of 50% ± 4% at a temperature of 23 ± 2 °C for curing. The length of the specimens was then measured at 7 days, 14 days, 21 days, and 28 days (L_x_). The extent of drying shrinkage was calculated as follows:

(4) Drying shrinkage = (L_i_ − L_x_)/G

where G is the nominal effective length, 250 mm.

Compressive strength was tested on 50 × 50 × 50 mm cube specimens in accordance with ASTM C109-13 [33]. Specimens were retrieved, dried, and tested at 7 days, 14 days, and 28 days to gauge compressive strength.

The UPV test was performed on cylindrical specimens with a diameter of 100 mm and height of 200 mm in accordance with ASTM C597-09 [34]. The measurement device used in this test was the Pundit Plus, manufactured by CNS Farnell Limited. Converters were placed at both ends of the specimens at 28 days, and the ultrasonic frequency was set at 54 kHz. Wave velocities were measured twice and then averaged to obtain the UPV value.

## 3. Results and Discussion

### 3.1. Properties of Fine Recycled Concrete Aggregates

Figure 1 presents the particle size distribution curves for FNA, R1, and R2, as well as the distribution range deemed acceptable in ASTM C33-13 [26]. The particle size distribution curves of all samples fell within the acceptable range. R1 was produced in a single stage crushing process and R2 was produced using multi-stage crushing. Thus, R2 contains a larger quantity of finer particles, which places the particle size distribution curve of R2 above that of R1.

**Figure 3:**
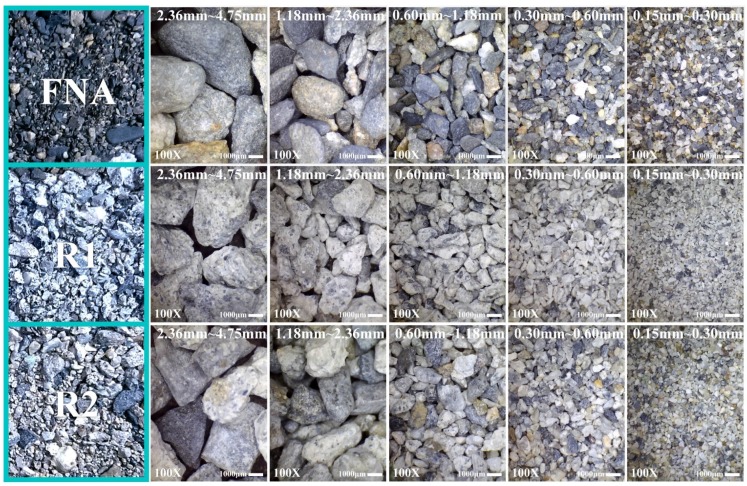
Comparison of appearances and particle size distributions of FNA, R1 and R2.

**Figure 4:**
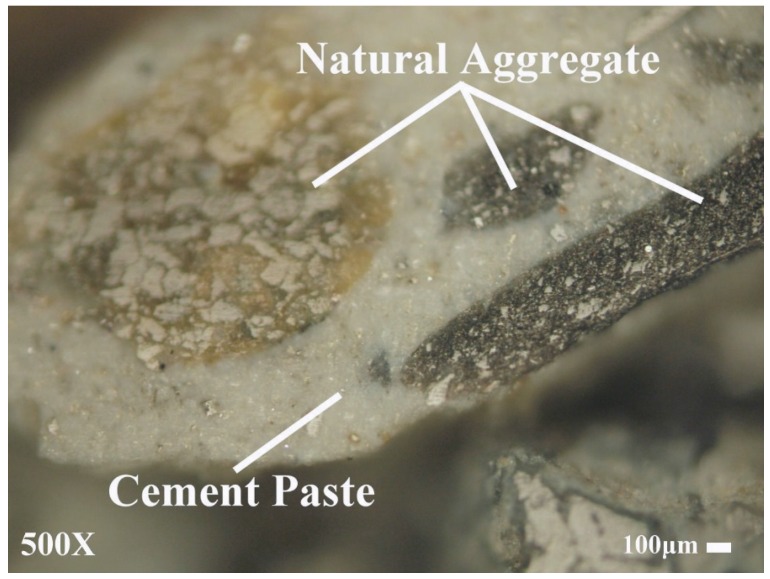
Microscopic observation of fine recycled concrete aggregates.

Figure 3 illustrates the appearance of FNA, R1, and R2. Clearly, particles in R1 are rougher in shape and more angular than those in R2 as well as more grayish in color. The difference in shape can be attributed to the repeated crushing and lack of coarse aggregate in the production of R2, such that it contains a higher percentage of fine aggregate. With regard to color, both materials were produced from concrete waste and thus had cement paste adhered to the larger fragments, as shown in Figure 4. We can therefore assume that the difference in color is due to a higher percentage of cement paste in R1 (more grayish in color) than that found in R2.

The attributes in Table 1 show that FRCAs (R1 and R2) have a lower density and a higher water absorption than does FNA. In addition, R1 has lower density and higher water absorption than does R2. The porosity of cement paste in FRCAs is higher than that of FNA; therefore, FRCAs the density is lower, and water absorption is higher [13,14,15,18,19,20,22,23,35]. As mentioned above, R1 contains a larger amount of cement paste than does R2 and therefore has lower density and higher water absorption. For the same reason, the fineness modulus of R1 exceeds that of R2, as shown in Table 1. These findings clearly demonstrate how the production process can influence the basic physical properties of FRCA.

### 3.2. Flowability

In accordance with the formulas in ASTM C1437-13 [30], we employed a flow table with a diameter 254 mm, such that the maximum flow value would be 154% (*i.e.*, (254 − 100)/100 × 100%). In addition, all of the mortar mixtures had a water/cement ratio of 0.55. Regardless of the amount of FRCA in the freshly mixed mortars, they all presented good flow values exceeding 154%.

**Figure 5:**
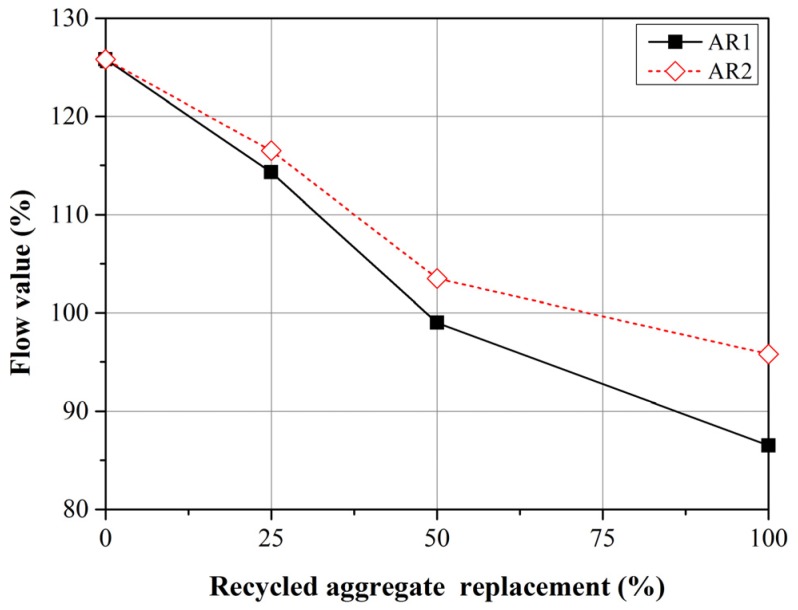
Flow values of mortar with W/C = 0.35.

Figure 5 presents the flow values of freshly mixed mortar with a water/cement ratio of 0.35. All of the mortar mixtures containing FRCA presented lower flow values than did the control groups. Furthermore, the flow values decreased with an increase in replacement ratio; *i.e.*, the proportion of FNA substitution with FRCA. With the same replacement ratio, samples that included R1 presented lower flow values than did those that included R2. This can be attributed to the fact that R1 fragments have a rougher surface texture and greater angularity, which increases the friction among the particles. It should be noted that all of the flow values obtained from mixtures with a water/cement ratio of 0.55 presented flow values exceeding 154% (data not shown).

### 3.3. Density

**Figure 6:**
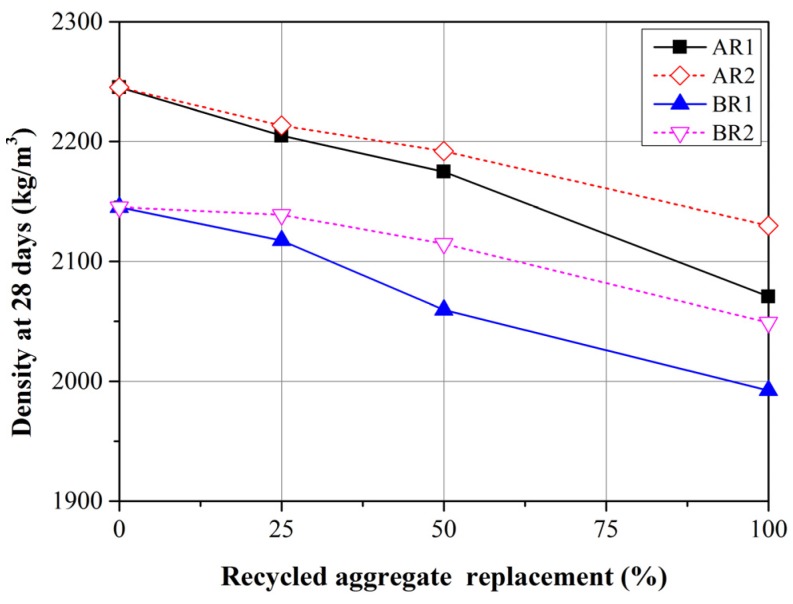
Density of mortars with R1 and R2 replacement at 28 days.

Figure 6 displays the mean density values from three mortar specimens at 28 days. All of the specimens containing FRCA had lower densities than did the control groups. In addition, the density of specimens containing FRCA decreased with an increase in replacement ratio. These findings are in agreement with those obtained in previous studies [14,16,18]. With the same replacement ratio, R1 samples present lower density values than do R2 samples. As shown in Table 1, the fact that the density of FRCAs is lower than that of FNA means that the density values of mixtures prepared using FRCA will also be lower. Increasing the replacement ratio also means that a higher proportion of FRCA leads to a reduction in the density of the mortar. The density of R1 is lower than that of R2; therefore with the same replacement ratio, the density of the mortars produced using R1 will also be lower than those produced using R2.

### 3.4. Absorption

**Figure 7:**
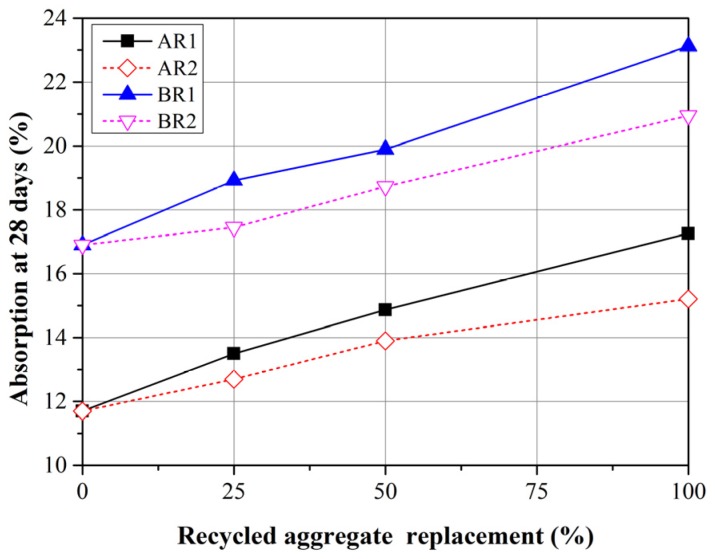
Absorption of mortars with R1 and R2 replacement at 28 days.

Figure 7 presents the mean water absorption from three mortar specimens at 28 days. The water absorption of the mortar mixtures with FRCA exceeded that of the control groups. Furthermore, the absorption increased with the replacement ratio. With the same replacement ratio, the absorption of mortar mixtures produced using R1 exceeded those produced using R2. This can attributed to the fact that the water absorption of FRCA is higher than that of FNA. These findings are in agreement with those of a previous study [13].

### 3.5. Drying Shrinkage

**Figure 8:**
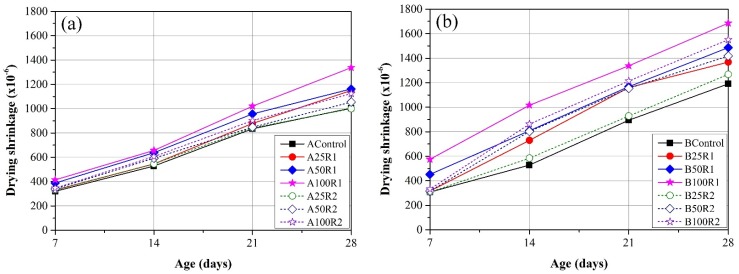
Development of drying shrinkage of mortars with (**a**) W/C = 0.35, and (**b**) W/C = 0.55.

Figure 8 present the mean drying shrinkage from four mortar specimens with water/cement ratios of 0.35 and 0.55, respectively, at 7 days, 14 days, 21 days, and 28 days. All of the specimens containing FRCA present higher drying shrinkage than did the control groups. This can be explained by the fact that the higher porosity of FRCA enables water to evaporate more rapidly. Increasing the replacement ratio also led to an increase in the drying shrinkage. With the same replacement ratio, the mortar containing R1 presented higher drying shrinkage than do the samples with R2. This can be attributed to the fact that the porosity of R1 exceeds that of R2.

### 3.6. Compressive Strength

**Figure 9:**
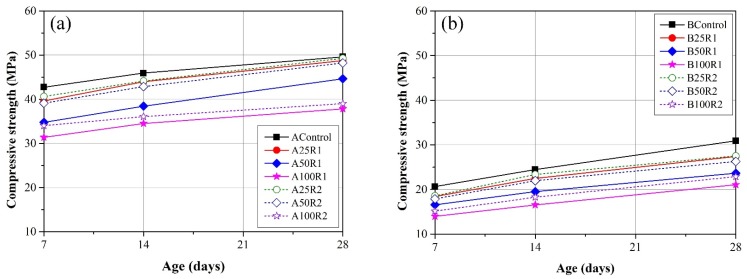
Development of compressive strength in mortars with (**a**) W/C = 0.35, and (**b**) W/C = 0.55.

The compressive strength of mortar specimens was tested at 7 days, 14 days, and 28 days. Figure 9 illustrates the development of compressive strength in specimens with water/cement ratios of 0.35 and 0.55, respectively. These values are the mean obtained from three specimens of each mortar mixture. The compressive strength of the specimens containing FRCA is lower than that in the control groups. This can be attributed to the fact that FRCA contains cement paste, which has greater porosity and therefore less compressive strength. Furthermore, compressive strength was shown to decrease with the replacement ratio of FRCA. These findings are in agreement with those obtained in previous studies [16,21]. In specimens containing the same proportions of R1 and R2, the R1 specimens presented less compressive strength due to the higher proportion of cement paste in the R1.

### 3.7. Ultrasonic Pulse Velocity (UPV)

**Figure 10:**
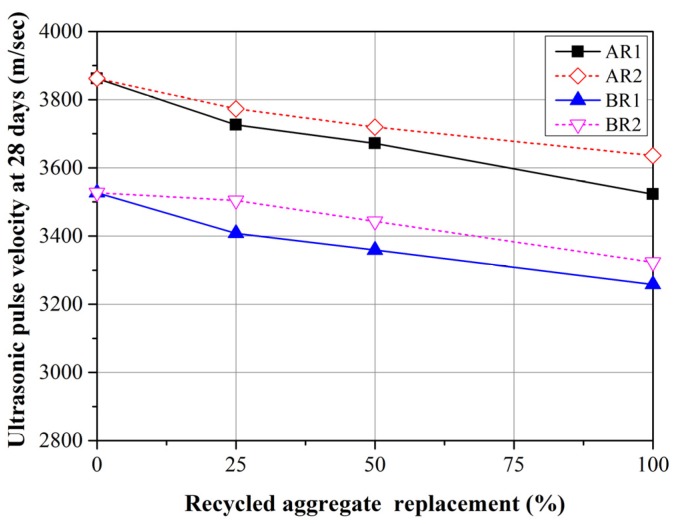
UPV values of mortars with R1 and R2 replacement at 28 days.

Figure 10 presents the mean UPV values from three mortar specimens at 28 days. All of the specimens containing FRCA presented lower UPV values than did the control groups and UPV values decreased with an increase in replacement ratio. These findings are in agreement with those obtained in [16]. Mortar containing R1 had lower UPV values than did the mortar containing R2. The porosity of mortars containing FRCA exceeded that of mortars containing FNA. Porosity inhibits the conduction of ultrasonic pulses, such that a higher FRCA content would lead to greater porosity, which would translate into lower UPV values.

**Figure 11:**
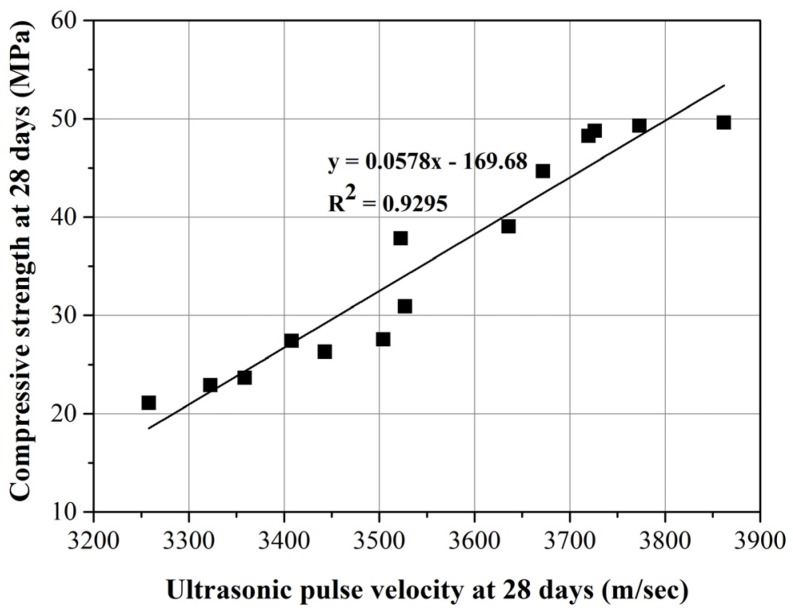
Correlation between compressive strength and UPV values of mortars at 28 days.

Figure 11 presents regression analysis of the UPV values and compressive strength of mortars at 28 days. The resulting regression formula with a R2 value of 0.9295 is *y* = 0.0578*x* − 169.68, where *x* and *y* denote the UPV value and compressive strength, respectively. Regression analysis results indicate that compressive strength increases with an increase in UPV value.

## 4. Conclusions

This study investigated the use of two types of fine recycled concrete aggregate (R1 and R2) obtained using different production processes at a recycling facility in Yilan, Taiwan. R1 was produced using a process that simultaneously produces coarse as well as fine aggregate by crushing concrete waste with a large jaw crusher. R2 was produced using a process that produces only fine aggregate through repeated crushing of concrete waste. We then tested mortar specimens in which various proportions of FRCA were substituted for FNA. This led to the following conclusions:

1. R2 has lower porosity, higher density, and lower water absorption than does R1, all of which are indicators of the superior quality of R2. This also demonstrates that the crushing process can significantly influence the quality of the resulting FRCA.
2. In all of the mortars containing FRCA, an increase in the replacement ratio led to a reduction in flow values, density, compressive strength, and UPV values. This is a clear demonstration that the replacement ratio is an important factor influencing the physical and mechanical properties of the resulting mortar.
3. When comparing mortars containing R1 or R2 at the same replacement ratio, the mortar containing R1 presented a lower flow value, lower density, higher absorption, higher drying shrinkage, lower compressive strength, and lower UPV values. This demonstrates that mortars containing R1 cannot match the physical or mechanical properties of R2, and further demonstrates the importance of the crushing process used in the production of FRCA.
4. Our results demonstrate the superiority of mortars produced using aggregate processed from recycled concrete via multistage crushing to obtain only FRCA. Nonetheless, the performance benefits of using only fine recycled concrete aggregate must be balanced against the additional energy requirements and subsequent impact on the environment.
